# Poly(methyl methacrylate) Coating of Titanium Workpieces to Reduce Burrs in Micro-drilling

**DOI:** 10.3390/mi10120838

**Published:** 2019-11-30

**Authors:** Luca Giorleo

**Affiliations:** Department of Mechanical and Industrial Engineering, University of Brescia, 25136 Brescia, Italy; luca.giorleo@unibs.it

**Keywords:** micro-drilling, burr reduction method, spin coating

## Abstract

A technique to reduce burr height in titanium micro-drilling is presented: a poly (methyl methacrylate) coating was applied before machining on the upper and lower surfaces of a titanium specimen (0.5-mm thick). After drilling, a cleaning process (acetone bath) was executed to eliminate the coating, and holes with less burr were obtained. The coating process was executed with a spin-coating machine. To test the efficacy of the technique, two different coating thicknesses (7.9 and 5.4 μm) and two drill bits (0.25- and 0.5-mm diameter) were evaluated. Qualitative and quantitative analyses of the holes obtained were performed with scanning electron microscopy and three-dimensional microscopy, respectively. The results highlight the efficacy of the technique to reduce the burr height by 70% in coated titanium relative to that in an uncoated titanium sheet.

## 1. Introduction

Manufacturing technologies for material removal have been constantly evolving to fulfill consumer requirements. Currently, downscaling of processes such as machining, injection molding, and electric discharge machining (EDM) is needed to produce microscale workpieces that are used in fields ranging from aeronautical to biomedical applications [1,2]. Despite the existence of several competing technologies, drilling is the most common and still in high demand. Frequently, the manufacturing of a through bore-hole is characterized by the formation of entrance and exit burrs. The dimension of the exit burr exceeds that of the entrance burr considerably. Owing to the hangover on the drill exit surface, the quality of the workpiece is reduced significantly. Generally, the dimensions of the burr are affected by the process parameters [3,4], and the height of the burr increases with the dimension of the drill bit diameter. Despite this, the problems associated with burrs became more critical when the environment shifts from macro- to microscale. Neugebauer, et al. (Figure 16 in Ref. [5]) reports the trend of the absolute and relative height (evaluated as the ratio between the burr height and hole diameter) as a function of the drill bit diameter.

As can be observed, the path of the absolute burr height shows an increase of the burr height in the range from 0.1 mm ≤ d ≤ 7 mm. Afterwards, the burr process mechanism becomes unstable for a change of burr geometry (from crown to ring). On the opposite, in the case of the micro drill bit with a diameter less than 2 mm, Figure 16 in Ref. [5] shows how the relative burr height exponentially increases [5]. In particular, when the drill diameter is lower than 1 mm, the relative burr height becomes higher than 1 which means the absolute burr height is higher than the hole realized.

In micromachining, this trend leads to a high amount of burr that surrounds the hole and affects its function. The exit burr degrades the quality of parts and is problematic for assembly, so a deburring process is necessary. Problems arise during manual deburring, which is often performed at a conventional size level and is difficult to apply. A chemical deburring process is often unsuitable because it can distort the shape of the hole. Moreover, burr generation is critical not only to industry but also to scientific applications. In micro-drilling, the dimensions of important output parameters are affected, such as hole diameter and roundness. These parameters are fundamental in tool wear analysis, but the burr makes it difficult to use a machine tool interferometer. In a previous study, the author tested a method to avoid this problem by choosing brass as a reference material to reduce burr interference in hole diameter measurement [6]. However, processes able to reduce burr are always preferred.

In the literature, several solutions have been studied to minimize burr. Referring to the process parameter, a linear correlation between the burr height and feed per tooth was found [7], in particular, benefits have been achieved imposing a feed variation model based on a sinusoidal function [8]. Gariani et al. reduced burr height controlling the cutting fluid quantity as a function of the heat generated [9]. Drill bit geometry still influences the burr generated and a direct correlation between burr height and cutting edge radius still exists [10]. da Silva et al. showed a considerable reduction in burr height when cutting tools with lower exit angles are used [11] and Sugita et al. proposed a new drill bit design to reduce burr in composite materials drilling [12]. Assisted machining is another solution able to reduce burr problem: Takeyama et al. demonstrated the efficacy of ultrasonic vibration during aluminum micro [13] and Hussein et al. found a relationship between high-frequency and drilling parameters to reduce burr height by 86% in Ti6Al4V drilling [14]. Pilny et al. used a vacuum clamping set up to obtain a reduction of approximately 50% in exit burr formation [15]. Despite the important results achieved in these studies, the proposed approaches to reduce burr formation by imposing new drill bit geometry, hybrid processing, or constraints such as feed rate or spindle speed are sometimes difficult to realize in industrial environments. In particular, the author wants to underline that the mentioned approaches as examples of ultrasonic-assisted machining or customized drill geometry have been tested with success but in a laboratory environment, and a real possibility to apply these methods in an industrial environment could be critical or impossible for production or plant constraints. On the contrary, strategies that could be applied in the pre-processing step could be investigated because they do not modify the drilling process.

Another approach that could be followed to reduce burr height is to apply an auxiliary material on the top and the bottom of the workpiece. Based on chip mechanism formation, burrs are formed at the entrance and exit surfaces of the workpiece and are due to tearing and bending actions followed by shearing or lateral extrusion; so the possibility to apply on these surfaces a material that could be removed after playing a significative role in protecting the surface form the press foot, driving the drill bit and improving the accuracy and in particular to reduce the burr height [16]. This technique is usually applied to improve position accuracy and reduce burr height in the drilling of printed circuit boards where an entry board and backboard are commonly used [17]. However, despite the benefits, the entry and backboard solution found application only in the case of 2 ½ D application because the auxiliary material is solid. Based on this idea, Kim et al. applied a soft material (cyanoacrylate adhesive) at the exit surface of a sheet to prevent the exit burr and achieved burr reduction in the drilling of low-hardness materials, such as aluminum or copper, but the technique proved to be ineffective for hard materials, such as AISI 304 stainless steel [18]. Starting from the last works to improve technique application, liquid coating was tested by the author; in particular, a preliminary study of a burr reduction technique to reduce burr height in titanium drilling is presented in which a biocompatible coating (PMMA) is applied to the upper and lower surfaces of a titanium sheet

To experimentally evaluate the proposed method, two thicknesses of coating and two different drills diameter were tested. The efficacy of the method in terms of burr homogeneity and height was analyzed with scanning electron microscope (SEM) and 3D digital microscopy, respectively. The results highlight some benefits and critical aspects of the proposed approach.

Of interest is the suitability of the method for application in biomedical device production. Titanium is strong, biocompatible, and completely resistant to bodily fluids, making it the material of choice for the manufacture of a myriad of medical and dental devices, such as artificial valves in the heart [19]; stents in blood vessels [20]; and replacement implants in shoulders, knees, hips, elbows [21] ears, and dental structures [22]. These devices often require machining, including micro-drilling. However, if the drilling process generates a significant amount of burr that cannot be removed, it may have an adverse influence on the survival of the implant and trigger adverse side effects in recipients; therefore, methods to reduce burr height play a fundamental role in medical device production, yet they must be developed with the constraint of using materials that do not contaminate the biocompatibility of the workpiece.

## 2. Materials and Methods 

### 2.1. Coating Process

**Figure 1:**
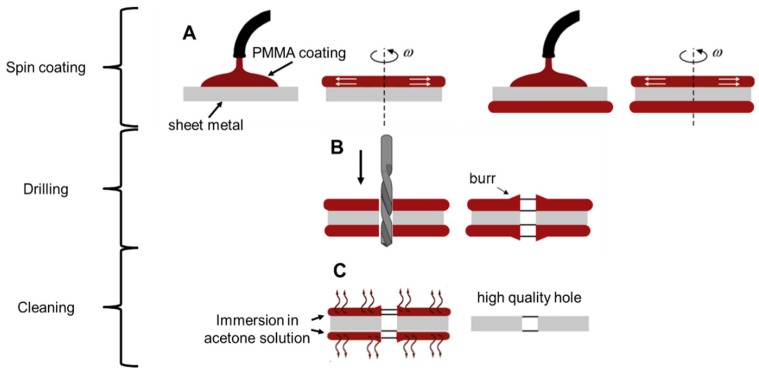
Scheme of the proposed method: coating step to apply extra material on the surface (**A**), drilling operation and relative burr formation (**B**), and chemical cleaning to remove coating and part of burr (**C**).

The top and bottom of the part to be machined are first covered with a film (step A), as shown in Figure 1. After machining, a cleaning process is executed to eliminate the coating, while avoiding surface microstructure modifications. Finally, a hole with a reduced top and bottom burr is obtained. The film should be thick enough to ensure that the burr is derived partially or completely from the deposited material (step B). The coating material is removed by a cleaning process (step C) to obtain very precise holes. Steps A and C are crucial to the feasibility of a precise machining process; therefore, parameters must be carefully identified to:

- ensure a low and controlled film deposition that contributes a negligible amount to the workpiece thickness;
- guarantee good adhesion between the substrate and the film to avoid detachment during the micro-drilling process;
- use a material that can be easily removed, avoiding any modification to the machined surface.

**Figure 2:**
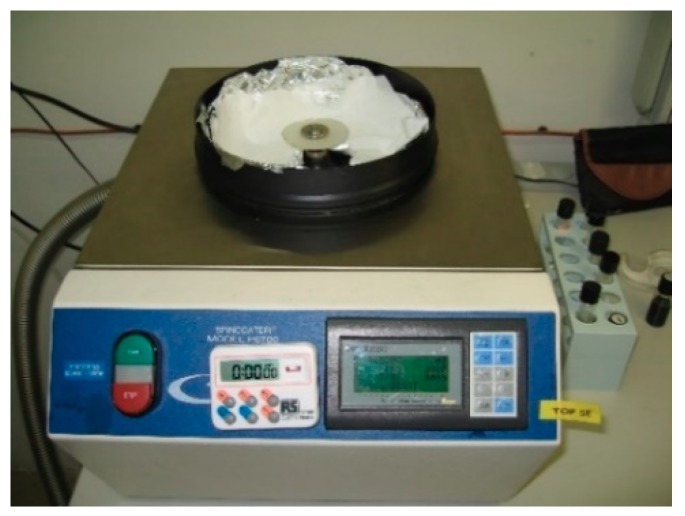
Spin-coating machine.

**Figure 3:**
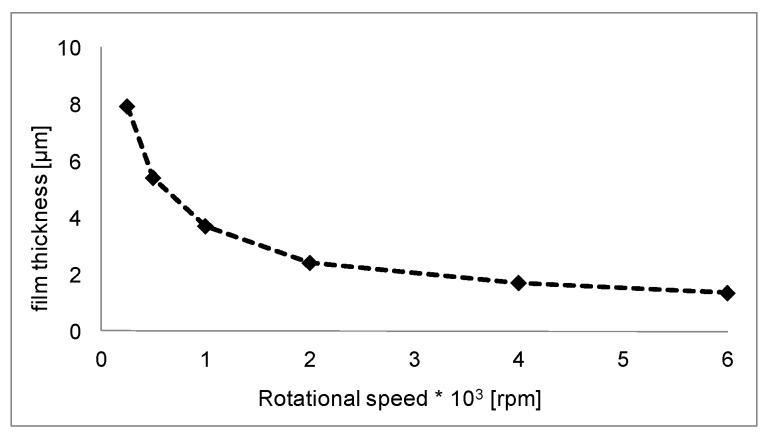
PMMA film thickness growth.

Based on these assumptions, spin-coating was selected as the deposition technique. Spin coating is widely used in science and engineering to deposit uniform coatings of organic materials and to uniformly distribute particulate matter on a flat surface [23,24]. In this process, the film thickness is a function of the material (in terms of solids content) and the revolution speed. The spin-coating technique deposits a thin and uniform coating (in the range of 0.1–10 μm). The thickness of a film *h* is given by Equation (1).

(1) $$h=\frac{KC^{2}}{f}$$

where *K* is a constant characteristic of the solvent-polymer system used, and *C* and *f* denote the concentration of the solution and the rotational speed (rpm) of the machine, respectively [25]. The coating selection focused on choosing a material that has not only good mechanical characteristics to support the machining process but also low chemical resistance for easy removal. We chose poly (methyl methacrylate), or PMMA, as the coating. PMMA swells and dissolves in many organic solvents, it also has poor resistance to many other chemicals due to its easily hydrolyzed ester groups. Nevertheless, its environmental stability is superior to many other plastics, such as polystyrene and polyethylene. Moreover, PMMA is extremely biocompatible and resistant to long exposure to temperatures, chemistry, and cellular activities in human tissue. In orthopedic surgery, PMMA is known as “bone cement” and is used to fill spaces between implants and bone because, in addition to its biocompatibility, PMMA is easy to make and manipulate in a hospital environment. In this study, a commercial Positive PMMA 950 k E-Beam Resists AR-P 630–670 series was used for the experiments, and PMMA was diluted in a chlorobenzene solution (9%) to produce the coating polymer. An SCS P-6700 top-precision spin-coating system was used to realize the coating (Figure 2). The film thickness of PMMA as a function of the rotational speed is shown in Figure 3. The film thickness decreases rapidly using speeds up to 1000 rpm until a thickness of 4 μm. To avoid depositing a film that was too thin, the authors tested the film deposition on two sheet metal coatings at rotational speeds of 250 and 500 rpm and obtained films that were 7.9 and 5.4 μm thick, respectively. Using the same process parameters, Ti grade 2 sheets were coated on the top and bottom surfaces as specimens to evaluate the burr reduction method. The sheet metal thickness was 0.5 mm. Specimen parameters are summarized in Table 1.

### 2.2. Micro-drilling Process

**Figure 4:**
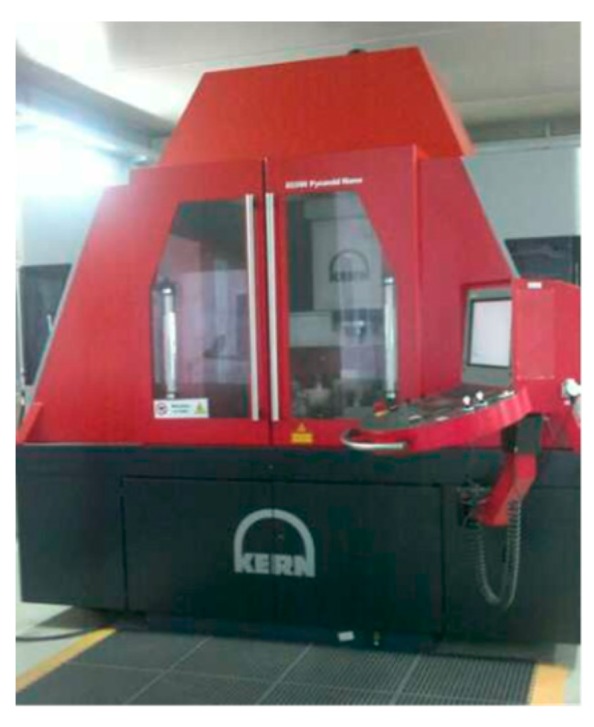
Kern Pyramid Nano.

**Figure 5:**
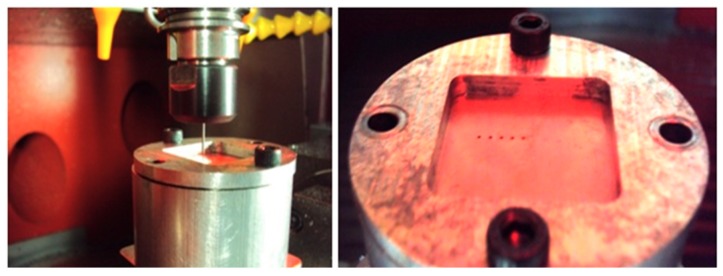
Clamping system.

Drilled holes were realized with a Kern Pyramid Nano, a five-axis ultra-precision CNC machine (Kern, Murnau, Germany), as shown in Figure 4. The clamping system shown in Figure 5 was created to avoid sheet movement during the drilling operation, and a gap of 5 mm was designed between the bottom of the sheet and the clamping system to allow for bottom burr generation without interference.

Two different diameters (0.5 and 0.27 mm) of HSS DIN 1899 drill bits (Sphinks, Derendingen, Switzerland) were used in the experimental campaign. The choice of the two drill diameters considered that, when moving from macro- to microscale processing, the drill’s core in proportion to the diameter is larger for smaller diameter tools in order to give them strength, which causes the chisel edge’s length to increase and influences the micro-drilling process reactions [26,27]. In other words, when the drill bit diameter becomes small, the process becomes more like a deep drawing process. If the hole generation process is different, the associated burr will also be generated with different dynamics. The selection of process parameters was set according to technical catalogs. To compare the effectiveness of the proposed method, three holes on the uncoated and coated sheet were executed for each drill bit diameter for a total of 18 holes (two diameters, three coatings, and three replicas). Table 2 summarizes the process parameters for each test specimen.

**Figure 6:**
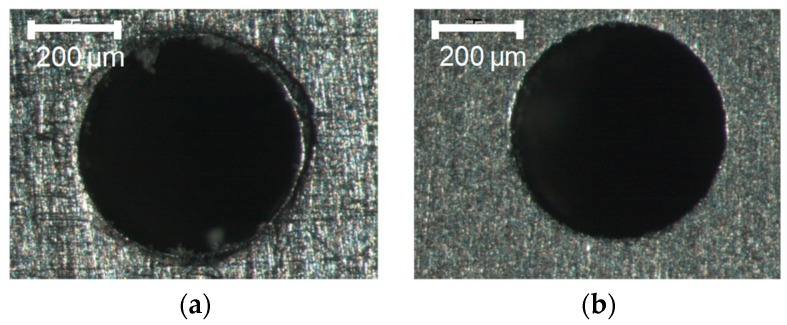
Drilled holes before and after cleaning. (**a**) After step B; (**b**) After step C

After drilling, the PMMA was removed by ultrasonic cleaning in an acetone bath for 10 min. The acetone chemically reacts with the PMMA and avoids any metal microstructure modification. Because it is possible for the ultrasound process alone to reduce the burr, uncoated sheets were also cleaned. As an example, images of a titanium sheet drilled with a 0.5-mm drill bit diameter are shown in Figure 6. After the cleaning treatment, a more precise hole is obtained.

## 3. Results

**Figure 7:**
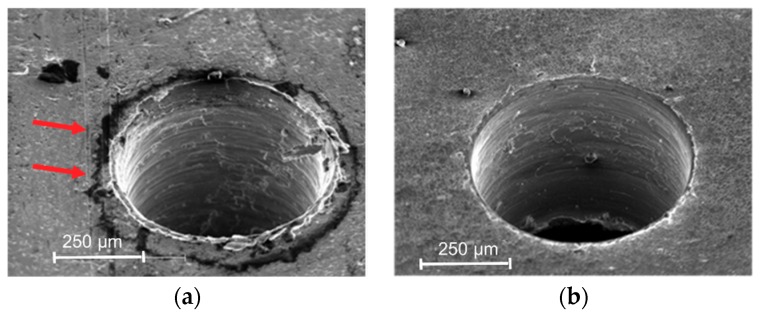
Localized presence or absence of PMMA (darker area) before and after the cleaning process, respectively. (**a**) Presence of PMMA; (**b**) Absence of PMMA.

To analyze burr homogeneity, images of the drilled holes before and after an ultrasonic cleaning in an acetone bath were acquired by SEM with a tungsten filament. The SEM analysis made it possible to establish the presence or absence of PMMA on the sheet after the cleaning operation due to a difference in electron beam absorption of PMMA that causes localized darker areas to appear (arrows in Figure 7). The results confirm the absence of PMMA from each cleaned titanium sheet.

**Figure 8:**
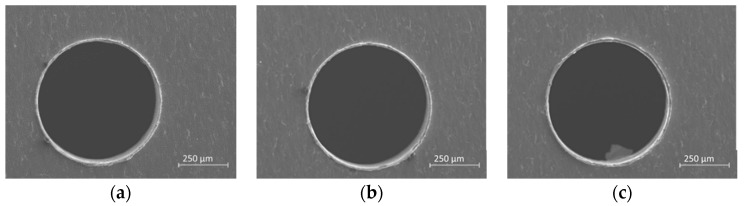
Three replicas of Test 6-ϕ 0.5-HI-Bottom view. (**a**) first (**b**) second (**c**) third.

The analysis of replicas reveals good repeatability of the experiments; a representative example is shown in Figure 8.

**Figure 9:**
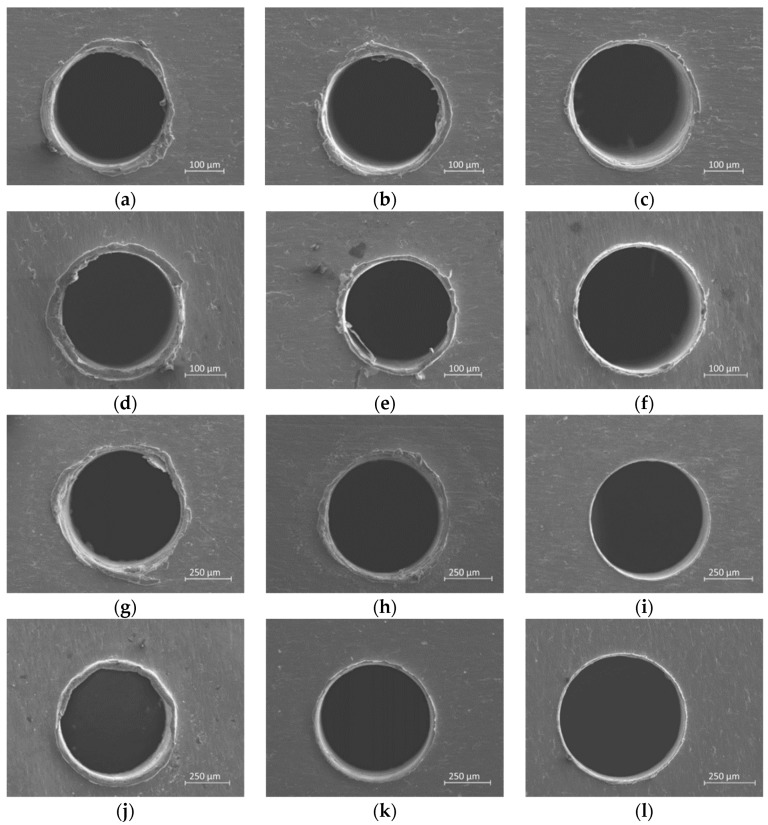
Results of qualitative analysis. (**a**) Test 1-ϕ 0.27-NO-Top; (**b**) Test 2-ϕ 0.27-LOW-Top; (**c**) Test 3-ϕ 0.27-HI-Top; (**d**) Test 1-ϕ 0.27-NO-Bottom; (**e**) Test 2-ϕ 0.27-LOW-Bottom; (**f**) Test 3-ϕ 0.27-HI-Bottom; (**g**) Test 4-ϕ 0.5-NO-Top; (**h**) Test 5-ϕ 0.5-LOW-Top; (**i**)Test 6-ϕ 0.5-HI-Top; (**j**) Test 4-ϕ 0.5-NO-Bottom; (**k**) Test 5- ϕ 0.5-LOW-Bottom; (**l**) Test 6-ϕ 0.5-HI-Bottom.

The experimental campaign results are shown in Figure 9. Images are labeled according to the following criteria: test number; drill diameter; coating thickness; and burr location. To better analyze the results, the coating thickness is classified as no thickness (NO), a thickness equal to 5.4 μm (LOW), and a thickness equal to 7.8 μm (HI); the burr is classified as either top or bottom.

Different assertions can be made from these results. First, for the 0.5-mm holes, there is a significant burr reduction between the coated and uncoated (Test 4 vs. Tests 5 and 6). Moreover, according to previous research [4] that established basic burr types in conventional and micro-drilling, there is a generation of transient burr for uncoated holes, while a uniform burr is generated for HI holes (Tests 1 and 2 vs. Tests 3 and 6). Finally, superior burr reduction is achieved for 0.5-mm holes (Tests 2 and 3 vs. Tests 5 and 6); the reason is attributed to the different drill bit geometry, such as chisel edge length, that affects the chip removal mechanism.

**Figure 10:**
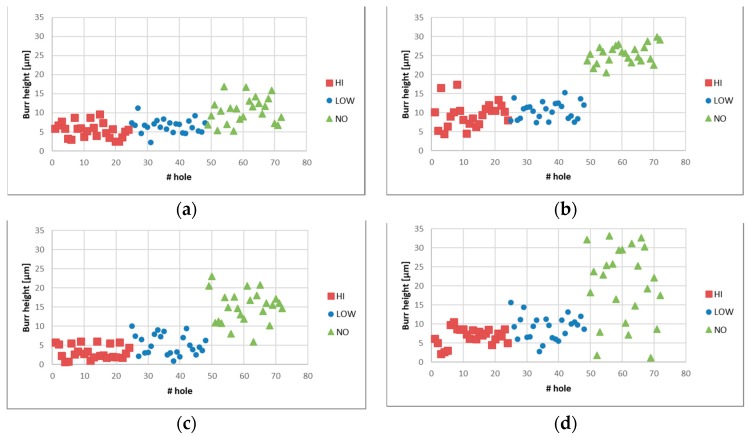
Burr height measurements as a function of the drill diameter (φ) and on hole position (Top/Bottom). (**a**) φ 0.27-Top; (**b**) φ 0.27-Bottom; (**c**) φ 0.50 Top; (**d**) φ 0.50 Bottom.

After SEM analysis, the burr height for each test was measured by 3D digital microscopy (Hirox). A 3D model of each hole was captured for each test, and eight height measurements were acquired. Figure 10 plots the results, and Table 3 summarizes the mean values of 288 burr height measurements (eight measurements, six tests, three replicas, top, and bottom).

**Figure 11:**
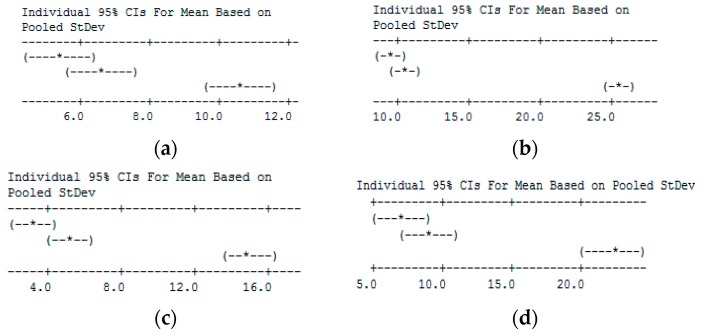
Results of Tukey’s range test. (**a**) ϕ = 0.27 mm-Top; (**b**) ϕ = 0.27 mm-Bottom; (**c**) ϕ = 0.50 mm-Top; (**d**) ϕ = 0.50 mm-Bottom.

A statistical approach was executed to analyze the results: a two-way ANOVA to verify the significance of coating and replicas and a Tukey’s range test to demonstrate whether the burr heights were significantly different from each other. The results of these statistical analyses are shown in Table 4 and Figure 11.

**Figure 12:**
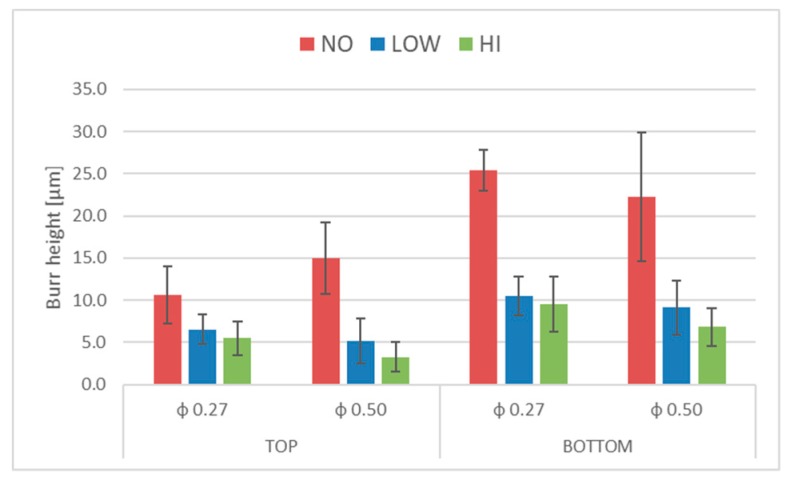
Mean burr heights relative to coating with standard deviations.

The ANOVA results confirm the efficacy of the technique (*p*-value of coating <0.05) and the robustness of the experimental design (differences among replicas are not significant). The results of the Tukey’s range test show that an improvement in burr reduction is achieved when a coating is applied, but the thicknesses of coatings tested yielded comparable burr heights. To better highlight the efficacy of the method, the mean height reduction and histograms with standard deviation are reported in Table 5 and Figure 12, respectively.

The following results are noted in Table 4 and Figure 12:

- Superior burr reduction is obtained for 0.50-mm holes compared with 0.27-mm holes. In particular, as reported in Table 4, the burrs generated on the top surface with the 0.5 drill diameter are reduced by 65% and 78% for LOW and HI coating thickness; on the contrary, a reduction of 38% and 48% is obtained with the 0.27 drill. Regarding the bottom surface, the burr height reduction is similar for both drills tested.
- The efficacy of the technique reduces bottom burr height by more than 50% in all tests, the minimum burr height recorded for top burr height is 38% (Table 4).
- A reduction in standard deviation is seen between coated and uncoated holes indicating that a more uniform burr is produced. This results is more evident in the case of bottom burr generated by the 0.5 drill diameter were a reduction of 50% of standard deviation is achieved (Figure 12).

In terms of the burr formation process, different considerations can be pointed out based on the qualitative and quantitative analysis. Figure 9 highlights that the coating affects the type of burr generated, in particular, in agreement with [28], an irregular burr is observed in holes realized without coating (Test 1 and 4). The effect of coating is to reduce the irregular trend when a low thickness of PMMA is coated during the drilling process (Test 2 and 5) and burr type becomes uniform in the case of high thickness (Test 3 and 6). Regarding the relative burr height problem that affects micro drilling, the authors also found in the case of titanium drilling results similar to what [5] reported in the case of steel; indeed, as reported in Table 3, the height burr is similar in the case of holes generated with a 0.27- or 0.5-mm drill diameter so that on the contrary the relative burr height is almost double that of the 0.27 drill bit. Finally, Figure 12 shows benefits in burr height reduction but the titanium burr is also still present in the coated sheets. As is known, the burr is formed by the plastic flow of the material and the role of the coating applied is to reduce/absorb this flow in order to only have a PMMA burr. The results highlight that the mechanical properties of PMMA coating are not able to fully compensate the pressure lift due to the titanium deformation so that a part of titanium burr is still generated. 

## 4. Conclusions

In this work, a new technique to reduce burr generation in titanium micro-drilling was tested. The technique consists of the deposition of a thin layer of PMMA on the top and bottom of a surface before the chip removal process. After, acetone cleaning was realized to remove the PMMA deposited. Spin coating was selected to realize uniform and thin coating. An experimental campaign was executed to measure the effectiveness of the technique with different drill diameters and coating layer thicknesses. The results highlight the efficacy of the technique, and burr height reductions between 40% and 80% were achieved. The founded results open the possibility to improve the surface quality of typical titanium medical devices (as such stents in blood, artificial knees, hips, elbows, dental applications) after a machining process without affecting their biocompatibility. In particular, nowadays different medical devices are produced using additive manufacturing processes that lead to the possibility of customizing the part design; however, additive process need post process machining operations to improve surface roughness or to realize micro features so that a technique able to reduce burr formation is needed. Future studies will be designed to understand the correlation between hole quality and coating thickness better.

## Figures and Tables

**Table 1:** Sheet metal-coated specimens.

Material	Composition (Wt %)	Dimension (mm)	f (rpm)	h (μm)
Ti grade 2	C: Max 0.1 Fe: Max 0.3 H: Max 0.015 N: Max 0.03 O: Max 0.25 Ti: 99.2	Length and width: 3 Thickness: 0.5	250	7.9
			500	5.4

**Table 2:** Process parameters used for the experimental campaign.

Test	Drill Diameter ϕ (mm)	Drill Tool Spindle Speed (rpm)	Feed (mm/min)	Coating Thickness (µm)
1	0.27	1705	5.11	0
2	0.27	1705	5.11	5.4
3	0.27	1705	5.11	7.9
4	0.5	2228	8.9	0
5	0.5	2228	8.9	5.4
6	0.5	2228	8.9	7.9

**Table 3:** Mean burr height measurements.

Drill Test	Top-φ = 0.27 mm			Bottom-φ = 0.27 mm			Top-φ = 0.50 mm			Bottom-φ = 0.50 mm		
Replica	HI	LOW	NO	HI	LOW	NO	HI	LOW	NO	HI	LOW	NO
1	5.9	6.5	9.9	10.0	10.4	23.9	3.3	5.6	14.6	6.0	9.9	22.2
2	6.4	6.9	11.2	7.7	10.4	26.0	3.3	4.6	14.8	7.5	7.1	22.7
3	4.2	6.3	10.8	11.0	10.8	26.2	3.3	5.3	15.6	6.8	10.4	21.9

**Table 4:** Results of two-way ANOVA.

Parameter	*p*-Value			
	φ 0.27		φ 0.50	
	Top	Bottom	Top	Bottom
Coating	0.001	0.001	0.001	0.001
Replica	0.36	0.157	0.876	0.931
Interaction	0.645	0.173	0.97	0.7

**Table 5:** Mean percentage of burr height reduction.

Hole Position	φ	LOW	HI
Top	φ 0.27	38%	48%
	φ 0.50	65%	78%
Bottom	φ 0.27	59%	62%
	φ 0.50	59%	69%

## References

[1] Masuzawa T. (2000). State of the art of micromachining. Cirp Ann..

[2] Ramsden J.J., Allen D.M., Stephenson D.J., Alcock J.R., Peggs G.N., Fuller G., Goch G. (2007). The design and manufacture of biomedical surfaces. Cirp Ann..

[3] Stirn B., Lee K., Dornfeld D. Burr Formation in Micro Drilling. Proceedings of the Sixteenth Annual Meeting of the American Society for Precision Engineering.

[4] Stein J.M., Dornfeld D. (1997). Burr formation in drilling miniature holes. Cirp Ann..

[5] Neugebauer R., Schmidt G., Dix M. (2010). Size Effect in Drilling Burr Formation. Burrs—Analysis, Control and Removal.

[6] Giorleo L., Ceretti E., Giardini C. (2011). ALD coated tools in micro drilling of Ti Sheet. Cirp Ann..

[7] Lee K., Dornfeld D. (2005). Micro-burr formation and minimization through process control. Precis. Eng..

[8] Lin T.R., Shyu R.F. (2000). Improvement of tool life and exit burr using variable feeds when drilling stainless steel with coated drills. Int. J. Adv. Manuf. Technol..

[9] Gariani S., Shyha I., Inam F., Huo D. (2017). Evaluation of a novel controlled cutting fluid impinging supply system when machining titanium alloys. Appl. Sci..

[10] Wyen C.F., Jaeger D., Wegener K. (2013). Influence of cutting edge radius on surface integrity and burr formation in milling titanium. Int. J. Adv. Manuf. Technol..

[11] da Silva L.C., da Mota P.R., da Silva M.B., Sales W.F., Machado Á.R., Jackson M.J. (2016). Burr height minimization using the response surface methodology in milling of PH 13-8 Mo stainless steel. Int. J. Adv. Manuf. Technol..

[12] Sugita N., Shu L., Kimura K., Arai G., Arai K. (2019). Dedicated drill design for reduction in burr and delamination during the drilling of composite materials. Cirp Ann..

[13] Takeyama H., Kato S. (1991). Burrless drilling by means of ultrasonic vibration. Cirp Ann..

[14] Hussein R., Sadek A., Elbestawi M.A., Attia M.H. (2019). Elimination of delamination and burr formation using high-frequency vibration-assisted drilling of hybrid CFRP/Ti6Al4V stacked material. Int. J. Adv. Manuf. Technol..

[15] Pilny L., De Chiffre L., Pìska M., Villumsen M.F. (2012). Hole quality and burr reduction in drilling aluminium sheets. Cirp J. Manuf. Sci. Technol..

[16] Huang X., Zheng L., Wang C., Wang L., Lin D., Liao B., Zhang L. Drilling Characteristic of Entry Board and the Influence on PCB Microdrilling Process. Proceedings of the 2016 11th International Microsystems, Packaging, Assembly and Circuits Technology Conference (IMPACT).

[17] Drilling and Routing Subcommittee (4–12) of the Fabrication Processes Committee (4-10) of IPC (2007). IPC-DR-572A Standard: Drilling Guidelines for Printed Boards.

[18] Kim D.W., Lee Y.S., Oh Y.T., Chu C.N. (2006). Prevention of exit burr in microdrilling of metal foils by using a cyanoacrylate adhesive. In. J. Adv. Manuf. Technol..

[19] Zhang L.-C., Chen L.-Y. (2019). A review on biomedical titanium alloys: Recent progress and prospect. Adv. Eng. Mater..

[20] Geetha M., Singh A.K., Asokamani R., Gogia A.K. (2009). Ti based biomaterials, the ultimate choice for orthopaedic implants—A review. Prog. Mater. Sci..

[21] Long M.M., Rack H.J. (1998). Titanium alloys in total joint replacement—A materials science perspective. Biomaterials.

[22] Gepreel M.A.H., Niinomi M.S. (2013). Biocompatibility of Ti-alloys for long-term implantation. J. Mech. Behav. Biomed. Mater..

[23] Burroughs J.H., Bradley D.D.C., Brown A.R., Marks R.N., Mackay K., Friend R.H., Burns P.L., Holmes A.B. (1990). Light-emitting diodes based on conjugated polymers. Nature.

[24] Mihi A., Ocana M., Miguez H. (2006). Oriented colloidal-crystal thin films by spin-coating microspheres dispersed in volatile media. Adv. Mater..

[25] Damon G.F. The Effect of Whirler Acceleration on the Properties of the Photoresist Film. Proceedings of the Kodak Seminar on Microemulsion.

[26] Klocke F., Gerschwiler K., Abouridouane M. (2009). Size effects of micro drilling in steel. Prod. Eng..

[27] Min S., Kim J., Dornfeld D. (2001). Development of a drilling burr control chart for low alloy steel AISI 4118. J. Mater. Proc. Technol..

[28] Kim J., Min S., Dornfeld D.A. (2001). Optimization and control of drilling burr formation of AISI 304L and AISI 4118 based on drilling burr control charts. Int. J. Mach. Tools Manuf..

